# Caoutchouc as a Remedy for Toothache

**Published:** 1845-05

**Authors:** 


*Caoutchouc as a Remedy for Toothache.*—Caoutchouc, becoming very smooth and viscous by the action of fire, has been proposed by Dr. Rolffs, as an excellent remedy for filling hollow teeth, and alleviating the toothache proceeding from that defect. A piece of caoutchouc is to be put on a wire, then melted at the flame of a candle and pressed, while warm, into the hollow tooth, and the pain will disappear instantly. The cavity of the tooth should first be cleaned out with a piece of cotton. In consequence of the viscosity and adhesiveness of the caoutchouc, the air is completely prevented from coming into contact with the denuded nerve, and thus, the cause of the toothache is destroyed.—*Braithwaite's Retrospect.*

